# Serotonin transporter (SERT) and translocator protein (TSPO) expression in the obese *ob/ob *mouse

**DOI:** 10.1186/1471-2202-12-18

**Published:** 2011-02-07

**Authors:** Gino Giannaccini, Laura Betti, Lionella Palego, Andrea Pirone, Lara Schmid, Mario Lanza, Laura Fabbrini, Caterina Pelosini, Margherita Maffei, Ferruccio Santini, Aldo Pinchera, Antonio Lucacchini

**Affiliations:** 1 Department of Psychiatry, Neurobiology, Pharmacology and Biotechnology, University of Pisa, University of Pisa, Via Bonanno 6, 56126 Pisa, Italy; 2 Department of Physiological Sciences, University of Pisa, Via delle Piagge 2, Pisa, 56124, Italy; 3 Department of Endocrinology and Kidney, University Hospital of Pisa, Via Paradisa 2, Pisa 56124, Italy

## Abstract

**Background:**

An ever growing body of evidences is emerging concerning metabolism hormones, neurotransmitters or stress-related biomarkers as effective modulators of eating behavior and body weight in mammals. The present study sought at examining the density and affinity of two proteins related to neurotransmission and cell metabolism, the serotonin transporter SERT and the cholesterol import-benzodiazepine site TSPO (translocator protein), in a rodent leptin-lacking mutant, the obese *ob/ob *mouse. Binding studies were thus carried out in brain or peripheral tissues, blood platelets (SERT) and kidneys (TSPO), of *ob/ob *and WT mice supplied with a standard diet, using the selective radiochemical ligands [^3^H]-paroxetine and [^3^H]-PK11195.

**Results:**

We observed comparable SERT number or affinity in brain and platelets of *ob/ob *and WT mice, whilst a significantly higher [^3^H]-PK11195 density was reported in the brain of *ob/ob *animals. TSPO binding parameters were similar in the kidneys of all tested mice. By [^3^H]-PK11195 autoradiography of coronal hypothalamic-hippocampal sections, an increased TSPO signal was detected in the dentate gyrus (hippocampus) and choroids plexus of *ob/ob *mice, without appreciable changes in the cortex or hypothalamic-thalamic regions.

**Conclusions:**

These findings show that TSPO expression is up-regulated in cerebral regions of *ob/ob *leptin-deficient mice, suggesting a role of the translocator protein in leptin-dependent CNS trophism and metabolism. Unchanged SERT in mutant mice is discussed herein in the context of previous literature as the forerunner to a deeper biochemical investigation.

## Background

The mechanisms of action of appetite hormones and related networks operating on body weight control have not been entirely established in mammals; the study of these pathways in models of mutant rodents, is contributing to elucidate the issue. In particular, the obese *ob/ob *mouse, mutant for the leptin (OB hormone) gene, is currently used to evaluate the pathogenesis of human obesity and type 2 diabetes: it lacks the functional leptin, develops hyperphagia, insulin resistance, hyperglycemia and hypercholesterolemia together altered immune response, impaired locomotor activity and fertility [1]. Leptin release from adipose cells is stimulated by insulin and glucocorticoids while counter-regulatory hormones inhibit its secretion [2].

In the hypothalamus, leptin exerts a double action: it up-regulates the release of anorexigenic neuropeptides as melanotropic hormone (α-MSH), CART (cocaine/amphetamine-regulated transcript) and CRH (corticotropin-releasing hormone) while inhibiting secretion of orexigenic neuropeptide Y (NPY), MCH (melanin-concentrating hormone), orexins, and AGRP (agouti-related peptide), all signals that, at the opposite, increases appetite and reduces energy consumption. Thus, *ob/ob *mice present a significant impairment of leptin-dependent hypothalamic pathways, accompanied by adrenal hypertrophy and increased corticosteroid secretion during diurnal rhythms [3,4]. The use of *ob/ob *animals has shown the involvement of catecholamines and serotonin (5-HT) in the leptin-deficient syndrome: in mutant animals, dopaminergic agonists reduce food intake and restrain metabolic dysfunctions, while SSRI antidepressant treatment decreases hyperphagia and hyperglycemia [1]. Some authors have observed a reduced expression of 5-HT transporter (SERT) mRNA in dorsal raphe nuclei of *ob/ob *animals accompanied by an altered locomotor activity [5]. Despite all these evidences, to our knowledge, there are still controversial opinions concerning the existence of interactions between leptin, 5-HT transmission and stress signals.

The aim of this study was therefore to investigate the expression of two pivotal proteins involved in 5-HT transmission and metabolism, SERT and the translocator protein (TSPO), in central and peripheral tissues of *ob/ob *mice in comparison with wild-type (WT) animals. SERT was evaluated given that it modulates the 5-HT re-uptake inside serotonergic neurons, a key mechanism permitting the activation/desensitization of 5-HT receptors within the synaptic cleft. SERT is also localized in periphery where it regulates platelet aggregation, gut peristalsis and immune response. An altered SERT has been reported in several complex human disorders, such as psychiatric diseases, pain and eating disturbances [6]. Density and affinity values of the TSPO protein were instead investigated as potential markers of stress-response and cholesterol metabolism in *ob/ob *obese mice. The TSPO protein is in fact the mitochondrial import of cholesterol with binding sites for benzodiazepines [7,8], also implicated in steroidogenesis [7-9]. The TSPO molecular complex is prevalently located on the mitochondrial membrane and is formed by: the main subunit, the isoquinolinic binding protein (18 KDa, identified as TSPO), the benzodiazepine binding portion VDAC (32 KDa, voltage dependent anionic channel) and the adenine nucleotide translocator ANT, which is also a target for benzodiazepines. Associated components of the complex are StAR (steroidogenesis acute regulatory) and PAP7 (associated protein 7) proteins, implicated in the steroidogenesis process [8]. Nonetheless, the precise function of TSPO is unknown [10]. TSPO has been indeed related to a variety of biological functions and processes, such as protection against reactive oxygen species, regulation of cell apoptosis, immunity and porphyrin transport [9]. Moreover, TSPO expression has been found changed in several diseases and pathological conditions [9], including psychiatric diseases and fibromyalgia where altered densities of both SERT and TSPO have been reported [11-13].

SERT and TSPO proteins were thus appraised by means of binding assays carried out in either neuronal or peripheral districts of *ob/ob *and WT mice. SERT was measured through the high-affinity SSRI ligand [^3^H]-paroxetine on brain and platelets, whereas TSPO was assessed by the isoquinilinic compound [^3^H]-PK11195 on brain and kidney. We also performed autoradiographic studies on limbic-hypothalamic coronal sections of mouse brain. Some previous works have in fact shown changes in SERT and 5-HT receptor subtypes at the level of the hypothalamus and limbic areas of mice fatten by a palatable diet [14,15]. Plasma chemical analyses were carried out in animals to evaluate their metabolic state.

## Results

### Analysis of blood chemical parameters

As shown in Table 1, *ob/ob *mice had higher levels of total cholesterol, glucose and High-Density-Lipoprotein (HDL) in comparison with WT mice. Concerning the other blood parameters examined, no relevant between-genotype difference was reported. γ-GT enzyme activity did not exceed 40 U lt^-1 ^(0-40 U lt^-1^) in *ob/ob *and mutant animals, indicating a normal hepatic function in all mice.

**Table 1:** Blood chemical parameters^(a) ^in mice.

Chemical parameters	*ob/ob*	WT
Glucose	158 ± 18	61 ± 6
Total Cholesterol	> 450	226 ± 19
High Density Cholesterol	87 ± 7	< 50
Triglycerides	27 ± 2	31 ± 4
Calcium	6.8 ± 0.4	6.1 ± 0.3

(a): Data are presented as mean ± SD (mg dl^-1^) of measures carried out in plasma samples separated from n = 3 *ob/ob *and n = 3 WT animals as indicated in the Methods section.

### [^3^H]-paroxetine and [^3^H]-PK11195 binding parameters

[^3^H]-paroxetine Scatchard analysis revealed a single population of high-affinity binding sites in all membrane preparations. [^3^H]-paroxetine equilibrium binding experiments (mean ± SD, 4 separate experiments in duplicate), showed no significant difference in either SERT density (B_max_, fmol/mg protein) or affinity (K_D_, nM) in both brain and platelets of *ob/ob *vs WT animals (Table 2).

**Table 2:** [^3^H]-paroxetine binding parameters^(a) ^in mouse membranes.

Brain:	Kd (nM)	**B**_**max **_**(fmol/mg protein)**
***ob/ob ***Mutant	0.1 ± 0.01	368 ± 17.5

Wild Type	0.09 ± 0.01	341 ± 18.0

**Platelets**:	**Kd (nM)**	**B**_**max **_**(fmol/mg protein)**

***ob/ob ***Mutant	0.09 ± 0.008	18680 ± 2278

Wild Type	0.08 ± 0.01	14830 ± 1722

(a): Data are presented as mean ± SD of n = 4 separate experiments in brain or platelet membrane preparations carried out in *ob/ob *and WT animals as indicated in the Methods section.

**Figure 1:**
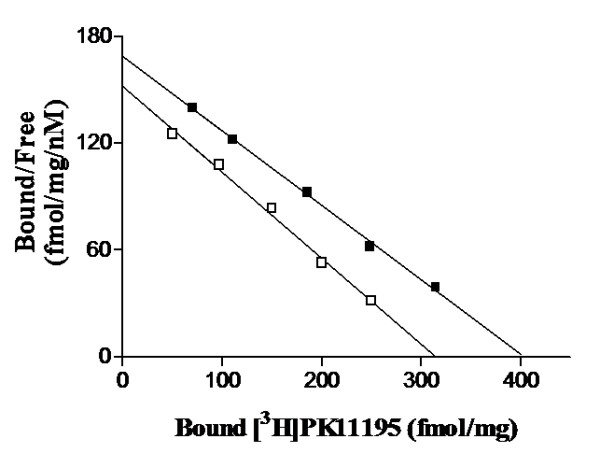
**[^3^H]-PK11195 binding assay in WT and *ob/ob *brain membranes**. Scatchard lines are representative of [^3^H]PK11195 saturation binding of 4 separate experiments carried out in brain membranes of obese *ob/ob *(□) and WT (■) mice.

[^3^H]-PK11195 binding Scatchard analysis also fitted with a single population of high-affinity sites in brain and kidneys. As shown in Table 3, no variation of [^3^H]-PK11195 binding affinity (K_D_, nM) was observed in brain and kidneys (mean ± SD, 4 separate experiments performed in duplicate) of different animals, whilst a significant increased B_max _was observed in the *ob/ob *brain (p < 0.05). Kidney B_max _resulted unchanged from comparison analysis of the two genotypes. Figure 1 depicts a representative linear transformation Scatchard, analysis obtained in brain membranes from *ob/ob *and WT mice, displaying an increased TSPO number in mutant animals.

**Table 3:** [^3^H]-PK11195 binding parameters^(a) ^in mouse membranes.

**Brain**:	Kd (nM)	**B**_**max **_**(fmol/mg protein)**
***ob/ob ***Mutant	1.75 ± 0.3	403.5 ± 12.6 (*)

Wild Type	1.8 ± 0.2	329.5 ± 16.0

**Kidney**:	**Kd (nM)**	**B**_**max **_**(fmol/mg protein)**

***ob/ob ***Mutant	1.9 ± 0.2	19550 ± 1180

Wild Type	2.0 ± 0.3	20260 ± 2017

(a): Data are presented as mean ± SD of n = 4 separate experiments in brain and kidney membrane preparations carried out in *ob/ob *and WT animals as indicated in the Methods sections. (*): Student *t-*test, p < 0.05.

### Autoradiography of brain coronal sections

**Figure 2:**
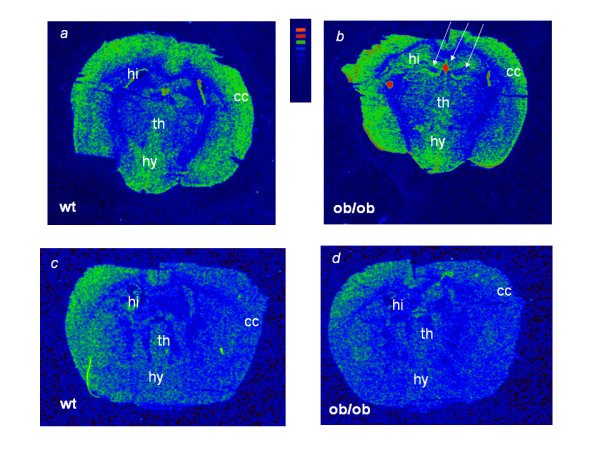
**[^3^H]-PK11195 (a,b) and [^3^H]-paroxetine (c,d) autoradiography in coronal hypothalamic-hippocampal sections of WT and *ob/ob *brain**. The increased signal corresponding to [^3^H]-PK11195 binding in sections of *ob/ob *mice is indicated by arrows (hippocampal region and choroids plexus-third ventricle). **cc**: cerebral cortex; **hi**: hippocampal region; **hy**: hypothalamus; **th**: thalamus.

Figure 2 depicts [^3^H]-PK11195 and [^3^H]-paroxetine autoradiography on coronal brain sections carried out at the hypothalamic-hippocampal level. TSPO binding sites labeled by [^3^H]-PK11195 (Figure 2.a,b) resulted unevenly distributed in these brain regions, with the highest expression in the cerebral cortex and hypothalamus. [^3^H]-PK11195 density signal was found increased in the dentate gyrus (hippocampus) of *ob/ob *mice (Figure 2b), together a binding raise in correspondence of peri-ventricular areas, at the level of the choroids plexus, especially that surrounding the dorsal third ventricle.

Figure 2 (c,d) also shows brain sections labeled by [^3^H]-paroxetine. These sections differed for about 55 μm from the [^3^H]-PK11195 ones (Figure 2.a,b), displaying a quite diffuse SERT labeling in cortex, hippocampus, hypothalamus and thalamus. Despite some decreased signal in the *ob/ob *cortex (Figure 2.d) vs. the WT one (Figure 2.c), these changes were not significant.

## Discussion

Since many years, the *ob/ob *mouse is conceived as a suitable experimental model for studying neuropeptide substrates and metabolic pathways implicated in human obesity and type 2 diabetes [1]: these rodents bear a nonsense mutation in the coding region of the leptin gene causing the lack of secretion of a functional peptide. An important aspect should be considered when using this mutant model: human obesity is rarely due to a single gene mutation [4] and obese humans prevalently present high circulating levels of leptin [16] together desensitized, leptin-resistant pathways; by contrast, *ob/ob *mice are defective in leptin production. Differences between these two conditions have not been still elucidated at the biochemical level. The *ob/ob *syndrome can be reversed by exogenous administration of leptin or by leptin gene transfection [1], proving that leptin receptors and networks are functional. On the other side, the study of *ob/ob *mice permits to dissect leptin-dependent neuroendocrine loops, involved in appetite control and energy balance dysfunctions. Indeed, as already mentioned, leptin pathways, co-modulation or connection with corticotropin releasing factor (CRF), NPY, α-MSH and neurotransmitter systems have not been fully characterized [2]. Specifically, energy expenditure balance and feeding behavior are regulated by redundant pathways: monoamines and leptin are both able to modulate food intake at the hypothalamic levels, but it is not precisely known if and how these signal molecules interact [17]. Some authors also consider leptin a signal evolved to prevent starvation rather than food plenty [18]. Beside alterations of glucose metabolism, another important feature of the *ob/ob *syndrome is the increased blood cholesterol in mutant animals. Cholesterol is the precursor of steroidogenesis, being a main, high-affinity ligand for the benzodiazepine site-translocator protein TSPO [7,9]. Reduced cholesterol levels have been evidenced inside macrophages of *ob/ob *mice, along with a diminished capacity in inflammatory response [19], supporting metabolic and hormonal cross-talks between immune response, inflammation and body weight signals [20,21]. Interestingly, platelet TSPO and SERT densities have been found altered in fibromyalgia [12,13], panic disorders [11,22] and suicide attempters [23,24]. This prompted us to preliminary assess the equilibrium-binding parameters of SERT and TSPO proteins, either in brain or high expression peripheral tissues, circulating platelets and kidneys, of *ob/ob *mice: to our knowledge, this is the first study that simultaneously evaluates SERT and TSPO expression in distinct anatomical district of a rodent genetic model of obesity.

Prior to SERT and TSPO analyses, blood chemical parameters were determined in *ob/ob *and WT animals to monitor cell metabolism: higher total cholesterol and glucose concentrations were observed in mutant vs. WT animals, according to data provided by the commercial source. Blood levels of the γ-GT enzyme were low in *ob/ob *and WT mice, indicating the absence of hepatic alterations. Moreover, *ob/ob *mice presented similar circulating triglyceride or calcium levels to those measured in WT animals. Concerning binding results, no difference of SERT density or affinity was reported in brain and platelets of *ob/ob *and control mice. Also, [^3^H]-paroxetine autoradiographic sections showed no appreciable binding differences between animals. This finding could signify that leptin-dependent pathways are altered in *ob/ob *mice without affecting 5-HT transmission. On the other hand, the fact that *ob/ob *mice show no changes in SERT density or affinity (present results) but a reduced SERT mRNA [5] is intriguing. Since leptin has been found able to decrease SERT binding sites in the rat brain [25], the *ob/ob *mouse could maintain the capacity at counteracting the decrease of SERT transcripts at the protein level, in the absence of SERT (or SERT-related) gene mutations. In fact, SERT underlies posttranslational regulation, trafficking or protein inactivation causing a differential distribution within cell compartments and/or discrepancies between mRNA and protein expression, as reported during megakaryoblastic differentiation [26]. Leptin-lacking mice could present an altered 5-HT responsiveness together a modified SERT reserve/function (uptake), without significant differences in binding sites. A deeper biochemical analysis of SERT should be therefore carried out, including flow cytofluorimetry, immunoprecipitation, gene expression, proteomic-functional (uptake) studies, in the context of 5-HT (or other monoamine) levels in blood and tissues of these animals. Concerning TSPO binding results, this protein was found increased in the *ob/ob *mouse brain while being similar in kidneys of mutant and WT animals. The [^3^H]PK11195 autoradiography of hippocampal-hypothalamic sections has revealed an up-regulation of TSPO density in two brain regions of *ob/ob *mice, the dentate gyrus of hippocampus and choroids plexus, indicating that TSPO number variations in *ob/ob *mouse brain are region-dependent. These results also underline that leptin chronic deficiency affects brain protein patterns.

The interpretation of our TSPO finding is difficult. In fact, to date, the precise role of TSPO within CNS is not understood. A brain region-specific regulatory mechanism in response to hypercholesterolemia and hyperglycemia could be active in these animals. Brain TSPO is mainly localized on glial cells and can be modulated by protein kinase C signaling [7] and the cAMP-protein kinase A pathway [9], activated by G-protein coupled receptors, including, therefore, metabotropic 5-HT receptor subtypes. In hippocampus and choroids plexus there could be an unbalance of regulatory signaling cascades, resulting in the enhancement of TSPO number. This could depend upon many factors, such as different receptor sub-type localization and activation: hippocampus and choroids plexus are brain regions at high expression of 5-HT_1 _and 5-HT_2C _receptors, respectively, and insulin inhibits choroids plexus 5-HT_2C _receptors [27]. Additional difficulties in interpreting our result come from the observation that increased TSPO has been associated with either tissue/neuronal damage or repair. Some authors have reported protective effects of TSPO agonists in experimental diabetic neuropathy [28], suggesting, hypothetically, reparative actions of brain TSPO in such a disease.

The same as for SERT, a deeper study of TSPO gene and protein expression together the investigation of its function and drug activities in *ob/ob *animals by means of different methodologies, including leptin treatment, is essential to confirm present results as well as to understand leptin-dependent neuronal trophism, metabolism and transmission. Another variable to consider is the different age of mutant animals: in our study, 4 month old mice were examined, when all symptoms of the "leptin-lacking syndrome" are present [1], but this does not exclude the diverse SERT and TSPO expression at other development or aging stages.

## Conclusion

Present results indicate that leptin-lacking mutant mice have an augmented density of TSPO in the hippocampus and choroids plexus, without changes of SERT protein number. Central TSPO variations are not paralleled by changes in periphery (kidney).

Despite limitations due to the small sample of animals investigated, our study indicates that the obese *ob/ob *mouse can be a challenging animal model to elucidate the mammalian leptin-dependent neuroendocrine-neurosteroid signaling involved in appetite control.

## Methods

### Materials

[^3^H]-paroxetine (specific activity, 24 Ci/mmol) and [^3^H]-PK11195 (specific activity, 69.9 Ci/mmol) were purchased from Perkin-Elmer Life Science (Milan, Italy). Hyperfilm MP were obtained from Amersham-Pharmacia (UK). All other reagents were obtained from normal commercial sources.

### Animals

5 *ob/ob *(C57BL6V-*Lep*^*ob*^) and 5 wild-type (C57BL/6J) adult (age: 15-17 weeks) male mice were obtained from Harlan (UK) and kept under standard laboratory conditions and feeding: animals were housed two per cage in saw-dust-lined cages at 22°C under a 12 h light/dark cycle, with free access to normal diet and tap water. All experimental procedures were carried out following the guidelines of the International European ethical standards for the care and use of laboratory animals (Community Council Directive 86-609). All protocols were approved by the Ethical Deontological Committee for animal experimentation of the University of Pisa.

### Sample preparation

#### Brain and kidney tissues

For experiments, all mice were sacrificed by cervical dislocation, in the morning, between 8.30-9.30 am, without modifying diet conditions or access to food. Kidneys and brains were rapidly removed and treated for membrane preparation and autoradiography. Brains were taken after removing cerebellum.

#### Blood collection for plasma chemical analysis

The day of sacrifice, blood was withdrawn by cardiac puncture of anesthetized mice and collected inside plastic tubes containing lithium-heparin to separate plasma. Blood was then centrifuged at 2000 g for 3-5 min at room temperature by means of a Micro-Centrifuge (Menarini, Italy). Glucose, total cholesterol, High Density Lipoprotein (HDL), triglycerides, calcium and γ-glutamil transpeptidase (γGT) were evaluated by an automated procedure. Briefly, plasma was automatically dispensed on a multi-layered reagent strip, specific for each dosage, and the resulting colorimetric reaction was analyzed by the automatic spectrophotometric analyzer SPOTCHEM SP-4410 (Menarini, Italy).

#### Blood collection for platelet separation

For separation of platelets used in [^3^H]-paroxetine binding assays, blood, withdrawn as above indicated, was kept in tubes containing 3% ACD (20 mM acid citric,110 mM sodium citrate, 5 mM dextrose) and centrifuged at 200 g for 15 min at room temperature. The supernatant, corresponding to platelet-rich plasma (PRP), was further centrifuged at 10,000 g for 10 min. The resulting pellet was washed by centrifugation in physiological solution at 10,000 g for 10 min and used for membrane preparation in [^3^H]-paroxetine binding assays (SERT).

### Membrane preparation

#### Preparation of cerebral membranes

To isolate cerebral membrane fractions, after sacrifice, brains (obtained as above indicated) from animals of each genotype (n = 4) were weighted, suspended in ice-cold 1:10 (w/v) 50 mM Tris-HCl, pH 7.4 (Tris-HCl buffer), containing 0.32M sucrose and protease inhibitors (200 μg/ml bacitracine, 160 μg/ml benzamidine, 20 μg/ml soy bean trypsin inhibitor), disrupted by means of an ultra-turrax homogenizer and centrifuged at 1000 g for 5 min at 4°C. Supernatants were then re-centrifuged at 48,000 g for 15 min at 4°C. For [^3^H]-paroxetine binding assay, resulting pellets were suspended in 1:10 volumes (w/v) of ice-cold Tris-HCl buffer, containing 120 mM NaCl, 5 mM KCl, protease inhibitors (as above indicated) and treated as previously described [29]. For [^3^H]-PK11195 binding, after the centrifugation step at 48,000 g for 15 min at 4°C, pellets were suspended in 1:10 volumes (w/v) of ice-cold Tris-HCl buffer without Na/K salts and washed twice in the same buffer by centrifugation at 48,000 g for 15 min at 4°C. Final brain membrane pellets were stored at -80°C until [^3^H]-paroxetine or [^3^H]-PK11195 binding assay.

#### Preparation of platelet and kidney membranes

For [^3^H]-paroxetine binding assay, platelet pellets (obtained as described previously), were suspended 1:10 (w/v) in 5 mM Tris-HCl buffer containing 5 mM EDTA and protease inhibitors, homogenized by ultra-turrax and treated as previously described [30] to obtain membrane-enriched fractions.

Kidney membranes for [^3^H]-PK11195 binding assays were prepared following a procedure slightly modified from Selleri and co-authors [31]. Briefly, tissue samples were diluted 1:10 (w/v) in ice-cold 50 mM Tris-HCl, pH 7.4, 0.32M sucrose, 1 mM EDTA (buffer A) containing protease inhibitors. After homogenization, tissue suspensions were centrifuged at 600 g for 10 min at 4°C. Supernatants (S1) were collected and kept on ice while pellets were re-suspended in the same buffer and re-centrifuged again as above indicated. The resulting supernatants (S2) were mixed to S1 and centrifuged at 6,500 g for 20 min at 4°C. Pellets were then suspended in 50 mM Tris-HCl, pH 7.4 (buffer B) homogenized and centrifuged again at 48,000 g for 10 min at 4°C. Final pellets, resulting from both procedures, were stored at -80°C until assay.

#### Protein assay

Total protein concentration was determined in brain, platelet and kidney membrane preparations according to the method of Bradford (Bio-Rad), using γ-globulins as the standard.

### [^3^H]-paroxetine and [^3^H]-PK11195 saturation binding experiments

[^3^H]-paroxetine binding (SERT) was carried out as follows [12]: membranes (50-100 μg protein/tube) were incubated with increasing radioligand concentrations (0.025-5 nM) in 50 mM Tris-HCl buffer, pH 7.4, containing 120 mM NaCl, 5 mM KCl (final volume, 2 ml). Non specific binding was assessed in the presence of 10 μM fluoxetine, used as the unlabelled displacer. All samples were assayed in duplicate and incubated 60 min at 22-25°C. TSPO binding was instead appraised as previously indicated [32]: brain and kidney membranes (25-100 μg protein/tube) were incubated with increasing concentrations of [^3^H]-PK11195 (0.5-8 nM), in a final volume of 0.5 ml assay buffer, 50 mM Tris-HCl, pH 7.4. Non specific binding was assessed in the presence of 1 μM PK11195 as the cold displacer. All samples were assayed in duplicate and incubated 90 min at 0°C.

In all binding experiments, incubation was halted by adding 5 ml of cold assay buffer. Samples were immediately filtered under vacuum through glass fiber GF/C filters and washed 3 times with 5 ml of assay buffer. Filters were then placed in pony vials with 4 ml of scintillation cocktail, and radioactivity measured by means of a β-counter (Packard LS 1600).

### [^3^H]-paroxetine and [^3^H]-PK11195 autoradiography on brain coronal sections

After animal sacrifice, the brain of *ob/ob *and WT mice was rapidly removed, placed in an embedding medium (O.C.T.), frozen in liquid nitrogen and stored at -85°C until use. Coronal sections (15 μm) at the hippocampal-hypothalamus levels obtained by cryostat were mounted onto gelatin-coated slides and stored at -30°C until assay. The day of assay, sections were allowed to equilibrate at room temperature for 10 min and pre-incubated in Tris-HCl buffer (50 mM, pH 7.4) for 15 min at 4°C. Sections were then incubated for: 1) 60 min in Tris-HCl buffer, containing 120 mM NaCl, 5 mM KCl, with 0.5 nM [^3^H]-paroxetine; 2) 90 min at 4°C in Tris-HCl buffer with 5 nM [^3^H]-PK11195 (TSPO). After incubation, all sections were washed twice with the Tris-HCl incubation assay buffer at 4°C for 30 sec, then with distilled water and rapidly dried under a cold air steam. Non-specific binding was determined by adding to the incubation solution an excess (10 μM) of unlabelled fluoxetine (SERT) or PK11195 (TSPO). Autoradiograms were generated by exposing tissue sections in tritium-sensitive films for 60 days at -85°C. Films were developed in Kodak D-19 for 5 min at 15°C and fixed in Kodak rapid Fix. After film exposure, tissue sections were fixed in 10% formalin and stained with hematoxylin-eosin for anatomical identification.

### Data analysis

Equilibrium-saturation binding data, the maximum binding capacity (B_max_, fmol/mg protein) and the dissociation constant (K_d_, nM) were analyzed by means of the iterative curve-fitting computer program EBDA and LIGAND [33]. Statistical analysis was performed by means of Student's *t *test (Graph pad version 3-4 program, San Diego, CA, USA). To improve visualization of [^3^H]-paroxetine and [^3^H]-PK11195 autoradiography, the gray scale, acquired by a X-ray scanner of autoradiographic films, has been translated into a color scale using the Image J software (Figure 2).

## Authors' contributions

GG, FS and AL conceived the study, participated in its design and coordination and helped to draft the manuscript. AlP participated in the study coordination and manuscript revision. GG, LP, LB, AnP and MM drafted the manuscript. LB, LS and ML carried out all radioligand binding studies. AnP carried out autoradiography experiments. LF and CP were responsible of tissue and blood sampling from animals and carried out blood chemical analyses. LB and LP elaborated experimental results.

All authors read and approved the final manuscript.

## Disclosure

The authors declare that they have no conflicts of interests.
